# Efficacy and safety of rituximab in patients with refractory neuromyelitis optica spectrum disorders: A prospective observation in Iranian cases

**DOI:** 10.22088/cjim.11.2.155

**Published:** 2020

**Authors:** Maral Seyed Ahadi, Abdorreza Naser Moghadasi, Nasrin Asgari, Mohammad Ali Sahraian

**Affiliations:** 1 Multiple Sclerosis Research Center, Neuroscience Institute, Tehran University of Medical Sciences, Tehran, Iran; 2 Institutes of Regional Health Research and Molecular Medicine, University of Southern Denmark; 3 Iranian Center for Neurological Research, Neuroscience Institute, Tehran University of Medical Sciences, Tehran, Iran

**Keywords:** Neuromyelitis optica spectrum disorders, Rituximab, Expanded disability status scale, Annualized relapse rate

## Abstract

**Background:**

Rituximab has been used successfully in the recent years for treatment of neuromyelitis optica spectrum disorders (NMOSD). However, a uniform treatment protocol for maintenance therapy and the best interval for evaluation and retreatment have not been postulated. We evaluated the efficacy and safety of rituximab treatment as second line therapy, in Iranian patients with refractory NMOSD, based on annualized relapse rate (ARR) and expanded disability status scale (EDSS).

**Methods:**

In this prospective before-after study, a total of 18 patients were treated with a loading dose of rituximab (375 mg/m^2^ weekly in 4 consecutive weeks). Flow cytometric determination of CD19^+ ^B cell in peripheral blood sample was carried every 6 weeks and patients were re-treated based on B cell repopulation with a single dose of 375 mg/m^2^. Wilcoxon signed rank test was used to evaluate the ARR and EDSS before and after treatment. A p-value of <0.05 was considered statistically significant.

**Results:**

Of the 18 patients, 10 (55.5%) were relapse-free during the period of follow up. The EDSS scores were reduced in nine (50%) patients and stable in the remaining nine (50%). The mean EDSS score before and after treatment were 4.1±0.4 and 3.7±0.3, respectively, which was statistically significant. There was also a statistically significant reduction in median ARR after treatment (1.48 (range 0.47-5) vs. 0 (range 0-2)). Rituximab administration did not have significant adverse effect in 94% of patients.

**Conclusion:**

Repeated treatment with Rituximab is an effective and well-tolerated treatment in refractory NMOSD.

Teuromyelitis optica (NMO) also known as Devic’s disease, was first described in 1894 by Eugène Devic as a syndrome characterized by acute myelitis and optic neuritis. During the past two decades, the definition and diagnostic criteria for NMO have evolved from Devic's clinical description. Discovery of a highly disease specific serum autoantibody against the astrocyte water channel aquaporin-4 (AQP4) in 2004 (1) and a broader clinical phenotype involving sites other than optic nerve and spinal cord has led to recognition of neuromyelitis optica spectrum disorder (NMOSD), diagnosed by 2015 criteria (2). Most patients with NMOSD have AQP4 targeted IgG1 autoantibody which targets astrocyte end feet surrounding the capillaries and pia matter. This leads to complement activation through the classic pathway leading to either lytic damage or may lead to activation of astrocytes and an inflammation due to NF-kB signaling. This could explain the preferential selectivity for the optic nerve and spinal cord, and to a lesser degree, the involvement of the area postrema and other circumventricular organs of the brain (3).

Based on the 2006 diagnostic criteria, the incidence rate and prevalence are estimated to be 0.053- 0.4 per 100000 person-year and 0.3- 4.4 per 100000 person, respectively (4-7). According to a new study in 2016, the prevalence of NMOSD in Tehran, capital of Iran, was estimated to be 0.86 per 100000 (8). Median age of disease onset, usually is in the late thirties, and tends to be a decade higher than MS; however there seems to be a vast range of age for disease onset, as about 25% of cases are in their childhood or fifties (9). NMOSD takes a relapsing course in more than 80% of cases with at least two relapses. Primary or secondary progressive course is observed in less than 2% of cases and the remainder constitutes the relapsing form of the disease. Therefore, the development of disability is attributable to aggregation of deficits, as the recovery tends to be incomplete in most cases which emphasizes the importance of relapse prevention (10). 

Immunosuppressive treatment consisting of azathioprine (11-13), methotrexate (14-16) and mycophenolate mofetil (13, 17) has been the mainstay of treatment in previous years. Regarding the discovery of Aquaporin-4 (1) and the role of humoral immunity in disease development, new treatment modalities have emerged. Rituximab, a chimeric anti-CD20 monoclonal antibody, targeting B cell population has been used successfully in recent years and repeated treatment courses with rituximab have been shown to be variously effective in different studies (18-20). 

However, a uniform treatment protocol for maintenance therapy has not been postulated. The most common reference for retreatment with rituximab is the detection of biomarkers CD19^+^ or CD27^+^ B cells in the peripheral blood mononuclear cells (18, 21-30). Nonetheless, there is not a consensus regarding the best interval for evaluation and retreatment. In this study, we evaluated the efficacy and safety of rituximab treatment as the second line therapy, in patients with refractory NMOSD, based on annualized relapse rate (ARR) and expanded disability status scale (EDSS), and proposed treatment protocol based on CD19^+^ B cell detection.

## Methods

**Patients: **This non-randomized prospective open label clinical trial was conducted between Aug 2014-2016 in Sina Hospital of Tehran, a Tehran University of Medical Sciences affiliated tertiary center. NMOSD was diagnosed based on 2006 diagnostic criteria (31) and were reevaluated according to 2015 diagnostic criteria (32). Patients would be included in the study if they had a previous treatment course consisting of an immunosuppressive for at least 6 months and had experienced an attack despite adequate treatment. Exclusion criteria consisted of prior or present history of malignancy, planning for pregnancy in the following 2 years, lactation, those in the reproductive age not willing to use contraceptives, chronic hepatitis or infections. Rituximab was provided as an off-label and unsponsored medication. Complete blood count (CBC), hepatitis B surface antigen (HBs Ag), anti hepatitis C virus antibody (anti-HCV Ab) and hepatitis B core antibody (HBC Ab) were checked at baseline and patients were excluded if proven to have chronic hepatitis. Informed consent was obtained from all the patients and the study was approved by the Ethics Committee of Tehran University of Medical Sciences (IR.TUMS.REC.1395.2667).

**Treatment Protocol and CD19 values: **Patients received an initial loading dose of 375 mg/m^2^ weekly in 4 consecutive weeks, based on pioneer studies (33, 34). Flow cytometric determination of CD19^+ ^and CD20^+^ in peripheral blood sample was carried out using Partec PAS flowcytometer, every 6 weeks, determining their percentage in lymphocyte gate. CD19^+^ B cell count > 1% of total lymphocytes in the peripheral blood sample was considered as B cell repopulation and a threshold for Rituximab administration (375mg/m^2^) as a maintenance therapy. 

**Clinical Assessment: **EDSS and ARR were evaluated at baseline and after the period of follow-up as the primary outcome of interest. Relapse was defined as acute or subacute emergence of neurologic symptoms, lasting at least 24 hours, in the absence of fever or infections, causing at least 0.5 points increase in the overall EDSS score at least 30 days apart from the last attack. 

**Statistics: **Wilcoxon signed rank test was used to evaluate the ARR and EDSS before and after treatment with rituximab. Kendall’s Tau-b was used to evaluate the correlation between EDSS difference and ARR ratio pre- and post- treatment with time to first attack and time to retreatment with rituximab. Time to first attack after treatment and time to rituximab re-treatment were assessed as secondary endpoints. A two-tailed p<0.05 was considered statistically significant. Statistical analysis was performed by means of statistical package for the social sciences (SPSS) Version18.0.0. 

## Results

**Demographic: **A total of eighteen patients (17 females) fulfilled the inclusion criteria and were registered in the study and were followed for a mean period of 12.7±2 months. The mean age at the beginning of rituximab administration was 32.7±2.1 years (range: 11-56). The mean duration of disease prior to rituximab administration was 6.5±1 (range: 0.7-16) years. 

Before rituximab, all patients had received at least one course of immunosuppressant, mostly combination of oral corticosteroid and azathioprine in 10 patients (55.5%) one of whom had also received mycophenolate mofetil, followed by azathioprine alone (5 patients, 27.7%) and prednisolone in one patient (5%). Nine patients (50%) had positive anti-AQP4 antibody and nine were seronegative for NMOSD. Rituximab was initiated if the patient had experienced an attack despite adequate treatment with immunosuppressant for at least six months. The mean EDSS score and the median ARR before treatment were 4.1±0.4 and 1.48 (range 0.47-5), respectively (table 1). 

**Table1:** Demographic, clinical and treatment profile of patients treated with rituximab

Case Number	Age	Sex	AQP4-Ab	ARR before treatment	ARR after treatment	EDSS before treatment	EDSS after treatment	Time to first attack, d	Time to Second Rituximab administration, m	Concomitant Disease	Age at onset, y/o	Disease duration, y	Total number of treatment cycles	Adverse Events	Immunosuppressive or Immunomodulatory
1	28	F^a^	Neg.	0.7	0	3.5	3.5	-	8	Single kidney	19	9	1	Immediate Allergic Reaction, Headache, Dizziness, Weight Loss	Mycophenolate Mofetil, Azathioprine Prednisolone
2	40	F	Pos.	4.56	2.00	6.5	6.5	-	2	Hypothyroidism, Hypertension, Diabetes	40	10	2	UTI, Back Pain, Shingles, Pruritus	Azathioprine Prednisolone
3	30	F	Neg.	1.78	0.87	6.5	6.0	95	4	-	14	16	3	Back Pain	Azathioprine Prednisolone
4	42	F	Neg.	0.61	0	4.0	4.0	281	4	IBS^d^	36	6	3	Pain, Flatulence	Azathioprine Prednisolone
5	35	F	Pos.	0.73	0	4.0	3.5	-	3	Minor Thalassemia,	28	7	2	Paresthesia, Back pain, Infection^a^,	Azathioprine Prednisolone
6	29	F	Pos.	0.96	0	2.0	2.0	-	2	-	21	7	2	Infection, Myalgia, Headache, Dizziness, Hair Loss, Palpitation, Throat Irritation, Genital Wart	Azathioprine
7	34	M^b^	Neg.	2.10	0	1.5	1.5	-	18	-	29	5	1	Throat Irritation, Paresthesia, Orthostatic Hypotension	Azathioprine Prednisolone
8	31	F	Neg.	2.06	0	2.0	2.0	-	2	Asthma, Hashimoto’s thyroiditis	29	1	2	Pain, Urticaria, Infection,	Azathioprine Prednisolone
9	31	F	Pos.	0.47	0	4.0	4.0	-	18	-	21	9	1	Back pain, Throat Irritation, Infection,	Azathioprine Prednisolone
10	56	F	Neg.	1.43	1.88	7.5	6.5	-	2	Diabetes, Hypertension,	53	2	2	Pain, Infection, DVT^e^	Azathioprine Prednisolone
11	11	F	Pos.	2.81	0	2.5	1.5	102	7	-	10	0	1	Weight loss, Urticaria	Prednisolone
12	30	F	Neg.	2.30	2.02	5.5	4.5	-	8	-	21	9	1	Pain	Interferon beta-1a, Azathioprine
13	39	F	Pos.	0.58	0	6.0	6.0	104	7	-	36	3	1	Weight loss	Glatiramer Acetate, Azathioprine
14	38	F	Pos.	1.54	0.94	3.0	2.5	-	6	Hypothyroidism	37	1	1	Infection	Azathioprine
15	31	F	Pos.	2.84	0.86	4.5	3.5	100	13	Hypothyroidism	17	13	1	Pain, Diarrhea, Anemia	Azathioprine
16	31	F	Neg.	1.29	0	4.5	3.5	385	4	Minor Thalassemia,	27	4	1	Pain, Leukopenia, Hair Loss, Arthralgia	Azathioprine, Prednisolone
17	32	F	Neg.	0.61	0.89	4.0	3.5	-	13	-	25	7	1	DVT, Headache, Dizziness, Pain, Back Pain	Azathioprine Prednisolone
18	22	F	Pos.	5.05	2	3.5	3.5	411	6	Hypothyroidism	21	1	1	Leukopenia, Infection	Azathioprine

F: female, M: male, AQP4-Ab: anti-aquaporin-4 Antibody, IBS: Irritable Bowel Syndrome, DVT: Deep Vein Thrombosis, UTI: Urinary Tract Infection, Pos: Positive, Neg: Negative, Y/O: Years Old, y: years, m: Months, d: Days

^a^: Infections constitute Upper Respiratory Tract Infections and Urinary Tract Infections

**Rituximab administration: **A total of 45 treatment courses were administered in our patients; the regimen used was 375mg/m^2^ weekly in 4 consecutive weeks (as the induction regimen), followed by 375mg/m^2^ (as the maintenance regimen). No other immunosuppressant was used concomitantly. All patients received premedication with intravenous (IV) steroids, anti-histamine and acetaminophen before each infusion. As previously stated, re-dosing was scheduled based on CD19^+^B cells re-population in the peripheral blood sample. There was no discontinuation of therapy; however there were increased intervals for CD19^+^ B cell count evaluation in some patients due to poor compliance.

**Treatment Efficacy: **Of the 18 patients, 10 (55.5%) were relapse-free during the period of follow up. The EDSS scores were reduced in nine (50%) patients and stable in the remaining nine (50%). A Wilcoxon signed rank test verified that at least one treatment course with rituximab did elicit statistically significant decrease in EDSS score (Z=-2.739, p=0.006) and ARR (Z= -3.506, p=0.000) in NMOSD patients. Median EDSS score ratings were 4 and 3.5 and median ARR were 1.48 and 0, pre-and post-treatment, respectively. The mean time to first attack was 191.6±50.9 days and the median time to second rituximab administration was 6 months (range 2-13). 

Kendall’s Tau-b was used to evaluate the correlation between differences in EDSS before and after treatment with time to first attack (τ=0.4, p=0.14) and time to retreatment with rituximab (τ=0.04, p=0.83) which were not statistically significant. Furthermore, correlation of ARR ratio before and after treatment with time to first attack (τ=-0.28, p=0.32) and time to retreatment with rituximab (τ=-0.06, p=0.73) were not statistically significant. A total of 12 relapses were recognized during the follow-up period, with patients’ number 2, 10, 12, 18 experiencing 2 attacks. Patient 2 had a 2-month delay in receiving the required maintenance treatment based on her previous lab data of CD19=1.4%. Her second attack occurred 9 days after a CD19=0.9%, which showed a rapid repopulation to 2 in 3 weeks. Patient 18 also experienced two attacks. In her first attack, she had a CD19 equaling 0.5% at the time of attack. The second attack occurred 9 weeks after her previous lab results of CD19=0.5%; Two weeks after her attack, the CD19 had incremented to 5. Patient 17 also had a 45-day-delay in rituximab administration and patient 15 had a six-week delay in obtaining the required laboratory tests. Four relapses in patients number 3, 10, 12 and 14 were associated with CD19 <1% within 2 weeks of their attack (A CD19 equaling 0.6 in patients number 3 and 14 and 0.2 and 0.1 in one of the attacks of patients number 10 and 12, respectively). 

**Adverse events: **Rituximab administration did not have significant adverse effect in 94% (17) of patients. The most common adverse events observed were minor infections, mainly urinary tract infection (UTI) and upper respiratory tract infections (URTI) (50%), generalized pain (38.8%), back pain (27.7%), headache, dizziness and throat irritation in 16.6% (table 2). One patient had immediate allergic reaction presenting with flushing, dyspnea and throat irritation mandating infusion cessation and corticosteroid administration. Infusion was resumed with a lower infusion rate when the symptoms resolved. The following courses of rituximab did not elicit allergic reaction. Two patients experienced transient leukopenia in the form of lymphopenia with spontaneous resolution and no major concomitant infection. There were no opportunistic infections; a patient experienced zona which was distributed in the mandibular territory of trigeminal nerve without involvement of contiguous dermatomes and another patient had developed genital wart which was managed with cryosurgery.

**Table 2:** Adverse events in patients treated with rituximab

**Adverse Events**	**Number of Patients (%)**
Infection (URTI, UTI)	8 (44.4)
Pain	7 (38.8)
Back Pain	5 (27.7)
Headache	3 (16.6)
Dizziness	3 (16.6)
Throat Irritation	3 (16.6)
Weight loss	2 (11.1)
Transient Leukopenia	2 (11.1)
Deep vein Thrombosis	2 (11.1)
Hair Loss	2 (11.1)
Urticaria	2 (11.1)
Acute Infusion related reaction	1 (5.5)
Genital wart	1 (5.5)
Chronic Diarrhea	1 (5.5)
Myalgia	1 (5.5)
Transient Anemia	1 (5.5)
Orthostatic hypotension	1 (5.5)
Shingles	1 (5.5)
Pruritus	1 (5.5)

URTI: Upper Respiratory Tract Infection, UTI: Urinary tract Infection

## Discussion

In this study we evaluated the efficacy and adverse events of rituximab as second line treatment in patients with refractory NMOSD and estimated ARR and EDSS pre- and post-treatment as primary endpoints. Re-dosing was planned based on re-emergence of CD19^+^ B cells. 

We observed a 100% reduction in median ARR and complete stabilization or reduction of EDSS following treatment with rituximab, which was in concordance with previously reported studies (18, 22, 35-38). Collongues et al. (39) evaluated the efficacy of rituximab as second-line treatment in refractory NMO patients and observed a 69.2% reduction in ARR and reduction of median EDSS from 5 to 3. The time from disease onset to rituximab administration in their study was 46±1 months, which was shorter compared to 77.5±12.4 months in our study. The lesser effect on ARR reduction in their study could be attributable to higher activity of the disease in the first years. However, the induction dose and the retreatment strategy were not stated. Mealy et al. also observed a 88.6% reduction in ARR in 30 patients with NMOSD treated with rituximab (40). However, they used different re-dosing strategy of CD19^+^ > 0.01% or fixed intervals of 6 months. Comparing their results to our retreatment strategy could emphasize the fact that higher thresholds for CD19^+^ could be practical with comparable effect and less cumulative dose of rituximab and perhaps fewer side effects.

The median time to second rituximab administration in our study, based on CD19^+^ B cell count, was 6 months (range 2- 13) which was in line with the speculated time of re-dosing in most prior studies (20, 30, 35, 36, 41). There is no known evidence regarding the accurate time of re-dosing in the literature. Greenberg et al. have reported the earliest return of CD 19^+^ B cells at 106 days after 1000 mg dose of rituximab, speculating that fixed 6 months intervals are not suitable for maintenance therapy (42). There are studies that have set CD19^+^ B cell count as a marker for specifying required time of treatment course, yet there are discrepancies regarding the suitable threshold for re-emergence of CD19^+^ B cells, with variable opinions from 0.1% (43, 44) to 1% (24, 26) and even 2% in some studies (42). Others have used a fixed interval of 6-9 months (33, 35, 36, 41) or a combination of methods, including expert opinion and clinical attack (18, 20, 27, 40, 45). There are other studies proposing repopulation of CD27^+^ memory B cells as the threshold for rituximab administration (21-23). However none are evaluated systematically to be the superior protocol and the heterogeneity of the protocols used inter-studies and intra-studies further add to the problem of reaching a consensus. Of the 12 relapses recorded during the follow-up period, four patients experienced two attacks. Two patients had a delay in receiving the required maintenance treatment, due to poor compliance, which resulted in an attack. Furthermore, patient 2 had a BMI equaling 32, which based on the study of Collongues (39) et al. could be a predictive of EDSS worsening, presumably due to the drug tissue distribution resulting in lowered effective dose. Her second attack occurred 9 days after a CD19 count of 0.9%, which later rapidly increased to 2% in 3 weeks. In two patients, attacks occurred 3 and 6 weeks after a missed appointment for obtaining the lab data (CD19), emphasizing the importance of checking the biomarkers at regular intervals. Five relapses occurred despite CD19<1% checked 2 weeks prior to the attacks. This could be indicative of either the need to consider lower thresholds for CD19 or other biomarkers predictive of relapse.

**Figure 1:**
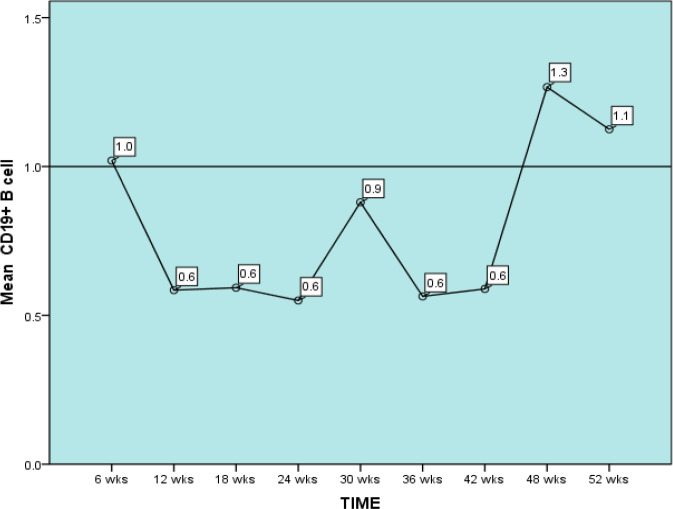
Mean CD 19+ B cell percentage over follow-up period. Wks: weeks

In a study to assess the effects of rituximab on lymphocytes in MS and NMO, Graves et al (46) reported an average decrease in B cells by 93% in 0-3 months, 81% at 4-6 months and 87% at 7-9 months after the first cycle of rituximab which were similar in the second and the third cycles. Furthermore, they reported a higher return of naïve B cells versus memory B cells and proposed that re-treatment does not affect as many memory B cells as the first treatment cycle. We also observed a mean repopulation of CD19+ B cells to 1 between 9.8- 11.2 months interval (figure 1). 

Nevertheless, Romero et al. demonstrated that in addition to relapses related to repopulation of CD27+ B cells, there are flare-ups which cannot be contributed merely to the presence of CD19+/CD27- or CD19+/CD27+, showing the importance of assaying both markers (47).

Rituximab was well tolerated in most of our patients except for one who developed infusion-related reaction. There was no rapid exacerbation following treatment and there were no major side effects mandating treatment cessation in the follow up period; the most common adverse events were non-opportunistic urinary tract infections and upper respiratory tract infections, followed by generalized pain which could be due to disease process itself. Safety profile of rituximab in our study was in line with other studies of NMO/NMOSD (38) with no malignancy or progressive multifocal leukoencephalopathy (PML) being identified during the follow-up period. There have been no PML reports consistent with rituximab administration in NMOSD patients to date. 

Our study was limited by the missing information due to the patients’ lack of cooperation for obtaining laboratory data, which makes it impossible to determine an association between CD19^+^ B cells and relapses. Future studies, with more regular testing of biomarkers for B cell repopulation can shed light on the aspect of individualized treatment with rituximab. In conclusion repeated treatment with rituximab, based on CD19^+^ B cell repopulation, is an effective and well-tolerated treatment in refractory NMOSD. However, other biomarkers may be applicable in evaluation of treatment in non-responders.

## Abbreviations: 

neuromyelitis optica spectrum disorders (NMOSD), annualized relapse rate (ARR), expanded disability status scale (EDSS), complete blood count (CBC), hepatitis B surface antigen (HBs Ag), anti hepatitis C virus antibody (anti-HCV Ab), hepatitis B core antibody (HBC Ab), progressive multifocal leukoencephalopathy (PML).
